# Inequitable coverage of vitamin A supplementation in Nigeria and implications for childhood blindness

**DOI:** 10.1186/s12889-019-6413-1

**Published:** 2019-03-08

**Authors:** Ada E. Aghaji, Roseline Duke, Ugochukwu C. W. Aghaji

**Affiliations:** 1 Paediatric Ophthalmology Unit, College of Medicine, University of Nigeria, Enugu, Nigeria; 2 Calabar Children’s Eye Centre, Department of Ophthalmology, University of Calabar Teaching Hospital, Calabar, Cross River State Nigeria; 3 Faculty of Arts and Science, University of Toronto, Ontario, Canada; 4 Faculty of Infectious and Tropical Diseases, London School of Hygiene and Tropical Medicine, London, UK

**Keywords:** Vitamin A supplementation, Childhood blindness, Nigeria

## Abstract

**Background:**

Vitamin A deficiency (VAD) is of major public health significance; it is a risk factor for childhood deaths from diarrhoea and measles in low and middle-income countries and an important cause of preventable childhood blindness in low income countries. Vitamin A supplementation (VAS) is being implemented in many LMICs and high coverage reduces the prevalence of blinding corneal diseases in children. However, national estimates of coverage may not reveal any inequities in intra country coverage. The aim of this study is to assess factors influencing VAS coverage and also assess the relationship between VAS coverage and childhood corneal blindness in Nigeria.

**Methods:**

Data were collected from the Nigeria Demographic and Health Survey (NDHS) 2013 and the published literature on population-based childhood blindness surveys in Nigeria. The main outcome measure was the proportion of eligible children who received VAS in the last 6 months preceding the survey. Study factors comprised a range of socioeconomic, and individual factors. Data were analysed using STATA V.12.1 (Statcorp, Texas). To explore the effects of the independent variables on VAS coverage, bivariate and multivariate regression was done. Variables with *p* < 0.05 in the final multivariable model were considered as independent factors. For the population-based childhood blindness surveys, aggregated and disaggregated data were used. Causes of blindness were stratified into corneal blindness and ‘others’. Odds ratios were computed to determine the odds of developing corneal blindness in each geopolitical region. Tests of significance were set at the 95% level.

**Results:**

The total VAS coverage in 2013 was 41.5%. VAS coverage was inequitable. Children with very educated mothers (OR 3.27 *p* < 0.001), from the south-south region (OR 2.38 *p* < 0.001) or in the highest wealth quintile (OR 2.81 *p* < 0.001) had higher odds of receiving VAS. The northwest zone had the lowest VAS coverage and the highest prevalence of corneal blindness.

**Conclusion:**

Regional and socioeconomic inequities in VAS exist in Nigeria and these may have grave implications for the causes of childhood blindness. The development and implementation of context specific and effective strategies are needed to reduce these inequities in VAS.

## Background

Vitamin A deficiency (VAD) is a major public health problem in low- and middle-income countries [1, 2]; It is a risk factor for under-five mortality from measles and diarrhoea [2] and is one of the most important causes of preventable childhood blindness in low income countries [3].

The primary cause of VAD is lack of an adequate intake of vitamin A. Children also have relatively high requirements for vitamin A, and demand for vitamin A increases during infections [3]. Globally, VAD affects an estimated 190 million preschool children [4] of which 56.4 million are in Africa [3]. A recent review indicated that although only 1.7% of the under-five mortality in low- and middle-income countries was attributed to VAD, 95% of these deaths occurred in sub-Saharan Africa and south Asia where 2% of deaths (same in both regions) were attributed to VAD [2]. In Nigeria, VAD is of severe public health importance, affecting over 20% of preschool age children. Strategies to prevent VAD include nutritional education to encourage dietary diversification and local production of vitamin A rich foods, food fortification with vitamin A, promotion of breastfeeding, the use of oral rehydration therapy to treat diarrhoea, higher measles vaccination coverage and vitamin A supplementation (VAS) [3]. It has been shown that VAS in pre-school children reduces morbidity and all-cause mortality in pre-school children [1].

VAS is recommended in all countries where the under-five mortality rate (U5MR) exceeds 70 deaths per 1000 live births [5]. In Nigeria the U5MR is currently 108/1000 live births [6], which is over four times the sustainable development goal target of 25 per 1000 live births [7]. VAS has been found to be one of the most cost effective strategies for improving child survival, hence it is being implemented as a child survival strategy in countries where VAD is of public health significance [4, 5]. To prevent VAD in many LMICs including Nigeria, vitamin A is delivered routinely to children aged 6–59 months as stipulated in the Integrated Management of Childhood Illness (IMCI) strategy at frontline health facilities, during bi annual Maternal Neonatal and Child Health Weeks (MNCHW) and National Immunisation Plus Days by trained healthcare workers [5, 8]. To maximize the potential of this intervention, it is crucial for countries to guarantee a high coverage of VAS. Coverage is an important implementation indicator, measuring the proportion of individuals who need the intervention that actually receive it [9]. VAS is being implemented in many LMICs and although coverage rates of up to 85% have been reported [10], VAS coverage is below this target in many countries including Nigeria [11]. However, national estimates of coverage may not reveal any inequities in intra country coverage. Analysis of subnational data will help to identify and assess within-country inequities in VAS coverage [9].

VAS reduces blinding corneal diseases in children. A systematic literature review and metanalysis of existing data shows that VAS in preschool children reduces corneal blinding lesions by almost 70% [12]. It is expected that countries or regions with high VAS coverage should have a lower prevalence of blinding corneal diseases. VAS coverage is recorded in regularly conducted national demographic and health surveys in Nigeria. In addition, population-based childhood blindness surveys have been conducted in three geo-political zones in Nigeria (Southeast [13], South-south [14] and Northwest [15] zones) and population-based estimates and causes of childhood blindness in these zones have been reported. The aim of this study is to assess factors influencing VAS coverage and also assess the relationship between VAS coverage and childhood corneal blindness in Nigeria.

The findings from this research will provide evidence-based information to policy makers and programme implementers on factors influencing VAS coverage and the relationship between VAS coverage and childhood corneal blindness.

## Methods

### Data sources

The data used were collected from the Nigeria Demographic and Health Survey (NDHS) 2013 and the published literature on population-based childhood blindness surveys in Nigeria. The Demographic and Health Surveys in Nigeria are implemented by the National Population Commission, with developmental assistance from partners such as the United Nations Population Fund (UNFPA) and the United Kingdom Department for International Development (DFID).

A steering committee is responsible for coordination and oversight and consists of high-level stakeholders from the National Bureau of Statistics, the Ministry of Health and the National Population Commission. The goal of the NDHS is to generate data to assist policy makers make informed policy choices and develop strategies for improving population health outcomes [16].

The NDHS is a nationally representative source of data on demographic and health statistics and collects information on child health characteristics, including maternal health factors, from a nationally representative sample of households. Sample selection was done by two -stage cluster sampling- first by selecting clusters from a list of enumeration areas, next by systematically selecting households from a list in the selected clusters using the 2006 census frame.

Data collection was by questionnaire. For the females, only women aged 15–49 were selected. Information sought included socio-demographic characteristics - age, religion, education, literacy, marriage, health characteristics, fertility preferences, antenatal, delivery, and postnatal care; breastfeeding and infant feeding practices, vitamin A supplementation, child immunisation and childhood illnesses. For mothers with children 6–59 months of age, data collected on relevant immunisations and vitamin A supplementation were based on mother’s recall and immunisation card where available. In addition, the household wealth index was calculated as a score of weighted household assets (such as ownership of vehicles or household facilities) and national level wealth quintiles were obtained. The response rate of women 15–49 years in the 2013 NDHS was 97.6% [16]. The detailed methodology can be found elsewhere [16].

Data sources for the childhood blindness surveys were from three population based childhood blindness surveys in three geopolitical zones in Nigeria; Northwest [15], South east [13] South-south [14] zones. These three studies used the key informant methodology to identify children suspected of having visual impairment, to determine population-based estimates and the causes of childhood blindness [17].

### Data extraction

The Children’s Data Recode File component of the data from the Nigeria Demographic and Health Survey (NDHS) for 2013 was retrieved as the database for this study. After reviewing the detailed data coding, further data cleaning was performed by the researchers. Data for 24,327 children aged 6–59 months were included in this analysis.

### VAS coverage indicator

For the NDHS survey, VAS coverage was defined as the percentage of children 6–59 months who received VAS within the 6 months preceding the survey [16].

### Study factors

Study factors comprised a range of socioeconomic, and individual factors, and their inclusion in the present analysis was based on findings from similar previously published studies in Sub-Saharan African countries like Guinea [18] Ethiopia [19] and a UNICEF summary of global VAS coverage [4] where an association was found between these variables and VAS coverage. These include age of the index child, urban/rural residence, geopolitical zone, mothers’ educational attainment (categorised as no schooling, primary or secondary and higher education), and household wealth index. The household wealth index was used to categorise households into wealth quintiles (as ‘poorest’, ‘poorer’, ‘middle’, ‘richer’ or ‘richest’) [16].

### Statistical analysis

Data were analysed using STATA V.12.1 (Statcorp, Texas). To allow for the cluster sampling survey design used in the NDHS, appropriate weights were used to restore the representativeness of the sample and analyses were performed using “svy” commands in STATA.

The main outcome measure was the proportion of children (6–59 months) who received at least one dose of Vitamin A in the 6 months preceding the survey. To explore the effects of the independent variables on VAS coverage, bivariate regression models were fitted separately for the 2013 NDHS data set. Subsequently, a multiple regression model was developed to assess the effects of variables found to be significant at *p* ≤ 0.05. These included socioeconomic, maternal education and regional variables. Models were restricted to children aged 6–59 months, living with respondents (eligible women aged 15–49 years). Both crude and adjusted odds ratios (OR) are presented with 95% confidence intervals. All tests were two-sided, and variables with *p* < 0.05 in the final multivariable model were considered as independent factors.

For the population-based childhood blindness surveys, causes of blindness were classified as corneal blindness and other causes. Odds ratios were computed to determine the odds of corneal blindness in each geopolitical region. Tests of significance were set at the 5% level.

## Results

The total VAS coverage for children 6–59 months in Nigeria in 2013 was 41.5%. There was no gender disparity in the coverage: females 41.8%; males 41.3% (Odds Ratio (OR) 1.02, 95% confidence interval (CI) 0.96–1.09, *p* = 0.47).

**Fig. 1:**
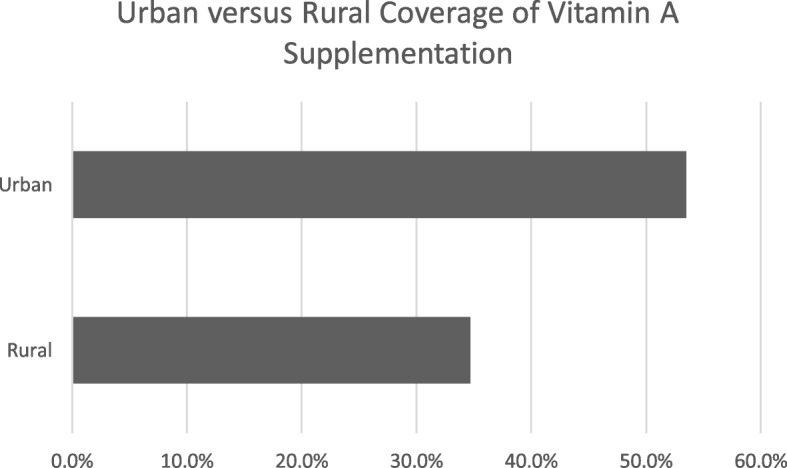
VAS coverage in urban and rural areas

VAS coverage was significantly higher in urban (53.5%) than in rural areas (34.7%) (*p* < 0.001), as shown in Fig. 1, and the crude OR for urban versus rural was 2.16 *p* < 0.001 while the adjusted OR was 0.87 *p* = 0.001.

**Fig. 2:**
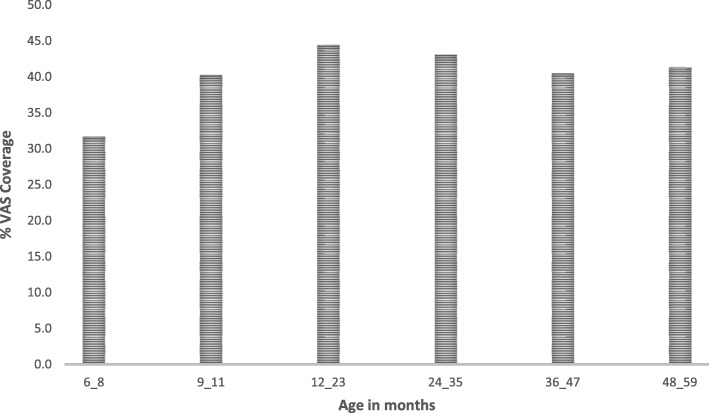
VAS coverage by age group

The lowest VAS coverage was in the 6–8 month age group (31.2%) as shown in Fig. 2. Using the 6–11 month age group as the reference population, the adjusted odds ratio for VAS coverage for all the other age groups was significantly higher (Table 1). The VAS coverage for children aged 12–59 months was 42.3%.

**Table 1:** Showing VAS coverage by child, maternal and sociodemographic characteristics

Variable	n	%	OR	95% (LCI-UCI)	p	Adjusted OR	95% (LCI-UCI)	p
Child Factors								
Age group								
6_11	3195	35.8	1			1		
12_23	5717	44.1	1.43	(1.29–1.59)	< 0.001	1.55	(1.39–1.73)	< 0.001
24_35	5111	43.1	1.35	(1.21–1.50)	< 0.001	1.46	(1.30–1.64)	< 0.001
36_47	5322	40.5	1.22	(1.09–1.35)	< 0.001	1.37	(1.23–1.54)	< 0.001
48_59	4982	41.3	1.26	(1.13–1.40)	< 0.001	1.42	(1.27–1.60)	< 0.001
Sex								
Female	12,042	41.3	1					
Male	12,285	41.8	1.02	(0.96–1.09)	0.47			
Maternal Factors								
Mother’s Education								
No education	11,198	25	1			1		
Primary	5005	47.9	2.75	(2.54–2.98)	< 0.001	1.66	(1.50–1.82)	< 0.001
Secondary	6560	59.6	4.41	(4.09–4.76)	< 0.001	2.02	(1.82–2.24)	< 0.001
> Secondary	1564	75	8.97	(7.76–10.37)	< 0.001	3.27	(2.75–3.89)	< 0.001
Sociodemographic Factors								
Residence								
Rural	16,030	34.7	1			1		
Urban	8297	53.5	2.16	(2.03–2.30)	< 0.001	0.87	(0.80–0.94)	0.001
Region								
North West	7566	26.1	1			1		
North East	4936	31.3	1.28	(1.18–1.41)	< 0.001	1.25	(1.14–1.37)	< 0.001
North Central	3644	45.5	2.36	(2.15–2.60)	< 0.001	1.56	(1.41–1.73)	< 0.001
South South	2930	65.5	5.37	(4.82–5.98)	< 0.001	2.38	(2.10–2.69)	< 0.001
South East	2190	56.7	3.71	(3.30–4.16)	< 0.001	1.76	(1.53–2.00)	< 0.001
South West	3061	65.3	5.32	(4.80–5.91)	< 0.001	2.3	(2.04–2.60)	< 0.001
Wealth Quintile								
Poorest	5324	21	1			1		
Poorer	5504	31	1.69	(1.53–1.86)	< 0.001	1.35	(1.22–1.50)	< 0.001
Middle	4859	43.3	2.86	(2.59–3.16)	< 0.001	1.72	(1.54–1.92)	< 0.001
Richer	4589	53.2	4.26	(3.85–4.71)	< 0.001	2.06	(1.82–2.34)	<0.001
Richest	4051	67.6	7.83	(7.03–8,71)	<0.001	2.81	(2.41–3.28)	<0.001

The North West zone had the lowest VAS coverage (26.1%) and South South had the highest (65.5%) as shown in Table 1. The level of mother’s education and wealth quintile were significantly correlated with VAS coverage in univariate and multivariable analyses.

The North West zone had the highest proportion of corneal blindness (affecting 55% of 20 children), which was significantly higher than in the South East zone (13% of 15 children) and South South zone (16% of 108 children) as shown in Table 2.

**Table 2:** Showing proportion of childhood corneal blindness by geographical region

Geopolitical zone	Corneal blindness		Other childhood blindness		Odds ratio	95% UCI-LCI	*p*-value
	n	%	n	%			
North West	11	55	9	45	1		
South East	2	13	13	87	0.13	0.02–0.71	0.02
South South	17	16	91	84	0.15	0.05–0.42	0.003

## Discussion

Our study shows that the VAS coverage for Nigeria (41.5%) is only half that recommended by the World Health Organisation (i.e., ≥80%) for children aged 6–59 months. This threshold is thought to be the minimum VAS coverage required for countries to achieve a measurable reduction in childhood mortality [5]. Our estimate is likely to be a minimal estimate as there is anecdotal evidence that during the MNCHWs, one mother may take several children to be immunized and receive vitamin A, including those from neighbouring households, without immunisation cards, so no formal records are made.

Our study showed no gender disparity in VAS coverage, similar to studies in Ethiopia and India [20, 21]. In some communities in northwest Nigeria and in Sierra Leone, it has been reported that VAS is not only facility based but also community based and health workers who adopt a door to door approach do not discriminate by gender [22, 23]. This may not be applicable to all communities as it has also been reported that VAS in Nigeria is mainly facility based [8]. However, a study in Ghana reported a higher female VAS coverage [24].

In our study, in the unadjusted model, the odds of urban children receiving VAS was twice that of their rural counterparts and an almost 20% difference in coverage. However, in the adjusted model, this was reversed with urban children marginally less likely to receive VAS. The higher crude odds could be explained by the higher number of uneducated and poorer mothers in rural areas. This is similar to research in India [20] and Ethiopia [19] where the crude odds ratio for VAS coverage showed higher coverage in urban areas, but this was reversed after adjusting for confounders. Our data show that the youngest age group ((6–11 months) had the lowest VAS coverage, similar to studies in Ethiopia [24] and Nepal [25]. It has been suggested that low coverage among children in this age group may be because some of the children aged 6–11 months would not have been eligible for VAS within the 6 months preceding the survey [25]. Our results support this hypothesis as the 6–8 month age group had the lowest VAS coverage. In a VAS coverage survey in Guinea, conducted 1 month after a VAS event, VAS coverage among 6–11 months old children was similar to children 12–59 months of age [18].

The National Strategic Plan of Action for Nutrition (NSPAN) provides an overview of the priority nutrition interventions and strategic directions for nutrition in the health sector in Nigeria, up to 2019 [26]. There is a bold and ambitious plan to increase VAS coverage in pre-schoolers to 95% by the end of 2018. However, the 2011 Multiple Indicator Report Survey showed a VAS coverage of 65% [27] while our results show a coverage of 41.5%. To achieve target coverage, there is an urgent need to address both the demand and supply side of VAS. Identifying inequities in coverage is an initial step to developing appropriate strategies to address them. We found evidence of socioeconomic and regional inequities in our study.

### Regional differences

The odds of a child in the South-south zone receiving VAS were almost two and a half times that of a child in the northwest zone. This zonal inequity has also been recorded in two previous NDHS in Nigeria [28, 29]. Wide intra country disparities in VAS have been reported in several countries [18–20]. In northern Nigeria which is predominantly muslim, cultural and religious beliefs may affect the uptake of health interventions, especially for maternal and child health [22, 30, 31]. It has been reported that issues pertaining to fathers’ approval significantly affect the uptake of immunisation or VAS in the northwest [22] and northeast [32] regions in Nigeria. However, the contribution of fathers’ approval to poor uptake of health intervention in the northern geopolitical zones needs to be interpreted with caution as the same may apply in other areas of the country where similar studies were not done. It was previously suggested that supply- side disparities in distribution of health facilities was a contributory factor for the health inequities between northern and southern Nigeria. However, recent research suggests that this is not the case and residents in the north have more physical access to vaccination centres than their southern counterparts suggesting that any disparities are likely due to socio-cultural differences [33]. This suggests that strategies to improve the uptake of health interventions should include targeted advocacy to community and religious leaders and male heads of households. A qualitative study on factors affecting the uptake of immunisation in Nigeria highlights the importance of sensitizing religious leaders especially in northern Nigeria [34].

### Mothers’ education and socioeconomic status

There is a growing body of literature that suggests that mothers’ education and socioeconomic status correlate directly with improved health outcomes and our study was no exception. In our study, the odds of the child of a mother with higher education receiving VAS was over three times that of the child of a mother without education. This is similar to results from Ghana [24] and India [20]. This may be because maternal education confers more awareness of the benefits of VAS. Research from Mali shows no disadvantage in VAS coverage for the children of uneducated mothers. The decentralised and context specific approach to disseminating health related information implemented in Mali may have reduced the inequity in access to health information [35]. Also, in a study in Sierra Leone, while data on socioeconomic and regional inequities are unavailable, high VAS coverage was achieved within clusters ranging from 86.7 to 97.8%. This was made possible by extensive and context specific social mobilisation [23]. This suggests that health promotion messages that are readily understandable by women without formal education need to be developed and scaled up.

The Africa Vaccination Week (AVW) is an initiative devised by the WHO AFRO to promote vaccination and ensure equity and access to its benefits. Several countries have used the Africa Vaccination Week to boost coverage of maternal and childhood health interventions, including VAS [36]. Nigeria’s equivalent of the (AVW) is the bi-annual Maternal New-born and Child Health Week (MNCHW) which started in 2010 and targets children under 5 years of age, pregnant women and women of child-bearing age with specific interventions. However, a recent evaluation of the initiative reveals that the current model of the MNCHW has failed to reach the most marginalized and there is no evidence that the MNCHW has significantly contributed to coverage of essential MNCHW interventions in Nigeria [8]. It has been suggested that failure of MNCHWs to reach the most marginalised populations in Nigeria is the because the bulk of interventions is facility-based and not community-based, and may not address the barriers of distance, opportunity costs and security [8].

This is reflected in our study which shows a low VAS coverage and high coverage disparities.

However, the NSPAN recommends that VAS be delivered not only in health facilities but also in community structures and campaigns/outreaches [26]. This has the potential to increase VAS coverage.

In addition, we can learn from the successful strategies of other African countries that have achieved high and equitable VAS coverage [18, 35] Such strategies include: developing context specific VAS strategies that are culturally and socially acceptable to the specific target audience [35], and maximizing the potential of the African Vaccination week by meticulous planning, coordination, supervision training and social mobilisation to ensure universal coverage for maternal and child health interventions [23].

Unless stakeholders in Nigeria develop and implement feasible strategies that target underserved and marginalised populations, achieving VAS coverage targets will be high on rhetoric and low on delivery.

### Implications of VAS coverage for childhood blindness

In many LMICs, corneal blindness from VAD and measles, which used to be the commonest cause of childhood blindness, is decreasing due to measles immunisation and VAS [37, 38]. In the southeast and south-south zones, corneal blindness was responsible for less than a sixth of all childhood blindness but in the northwest zone with the lowest VAS coverage, over half of children were blind from corneal scarring. Corneal blindness from VAD or measles is a reflection of individual (care giver), community and systemic lapses; there is need for care givers and community members to be sensitised about the importance of child health interventions and the consequences of non-compliance; it is crucial for health workers to understand the importance of VAS and measles immunisation [39]; finally, it is imperative that health systems deliver effective strategies to resolve both demand and supply side constraints to providing universal coverage with child health interventions.

### Limitations

In interpreting the results of this study and other NDHS studies, a few methodological limitations and strengths should be taken into consideration. The major strengths of the study are its national representativeness, large sample size and the availability of individual and household level factors which could influence VAS coverage. A major limitation is that the data on VAS available for analysis did not indicate whether data came from observation of children’s immunization cards, or from recall by mothers. The latter may be subject to recall bias depending on the interval between VAS and the NDHS survey.

Finally, analyses of the childhood blindness data should be interpreted with caution as these are population-based estimates derived from studies with small sample sizes and the data may not be normally distributed. In addition, the key informant method has limitations, and may not identify all children who are blind in a given population. However, due the challenges in the epidemiological investigation of relatively rare diseases like childhood blindness, this method has been used to provide population-based estimates of rare diseases and disability [17].

## Conclusion

Many studies have shown that VAD increases the risk of childhood mortality and this can be prevented by VAS. Regional and socioeconomic inequities in VAS exist in Nigeria and these may have grave implications for the causes of childhood blindness. The development and implementation of context specific and effective strategies are needed to reduce these inequities in VAS.

## Abbreviations

AVW: African Vaccination Week
MNCHW: Maternal Neonatal and Child Health Week
NDHS: National Demographic and Health Survey
OR: Odds ratio
U5MR: Under five mortality rate
UNICEF: United Nations Children’s Fund
VAD: Vitamin A deficiency
VAS: Vitamin A supplementation
WHO AFRO: World Health Organisation, Africa Region
